# Regenerated Fibers from Rennet-Treated Casein Micelles during Acidification

**DOI:** 10.3390/gels9070538

**Published:** 2023-07-03

**Authors:** Ronald Gebhardt, Novin Darvishsefat

**Affiliations:** Chair of Soft Matter Process Engineering (AVT.SMP), RWTH Aachen University, 52062 Aachen, Germany

**Keywords:** casein fiber, swelling, milk gels

## Abstract

Micellar casein fibers of defined size and internal structure can be produced by the extrusion of cold-renneted casein micelles into a warm, calcium-rich coagulation bath. Calcium phosphate contacts within the casein matrix are important for fiber stability and production but become less important under acidic pH conditions. We demonstrate this with swelling experiments in media with pH < 2, which we adjust with citric acid of different molarities. In contrast to the simple swelling of dried casein fibers in water, a two-phase process takes place in citric acid similar to swelling in 1 N HCl. However, instead of a second deswelling step, we observe in citric acid that the fiber swells further. The observation is explained by a pH-dependent transition from a rennet casein gel to an acidified rennet gel. This can be simulated with a kinetic model that couples two second-order rate equations via a time-varying ratio. The final swelling values decrease with increasing proton concentration via a scaling relation, which is also confirmed by swelling in other acids (HCl or acetic acid) in this pH range. We attribute this to a decrease in the molecular weights of the aggregated casein structures within the strands of the gel network.

## 1. Introduction

Rennet and acidified milk gels are the basis for the production of fermented milk products and differ fundamentally in the nature of stabilizing interactions, e.g., in the existence of calcium bonds [1,2,3]. While the gelation process has been studied with a number of experimental high-resolution methods and described with theoretical models [4,5,6], structural changes within existing gel networks have been studied in much less detail. Recently, we reported on the structure of micellar casein fibers with an internal structure formed by a particle gel network [7]. This is composed of a total of four different polypeptides (α_S1_-, α_S2_-, β-, and κ-casein), which together form the main protein component of milk. Caseins have an open and flexible conformation without a defined secondary and tertiary structure [8]. Caseins can be structurally described as diblock copolymers, as they consist of clusters of hydrophilic phosphoseryl groups as well as hydrophobic amino acids [9,10]. They form together with colloidal calcium phosphate spherical association colloids (called ‘casein micelles’ for historical reasons) with diameters distributed between 80–400 nm [11]. Within the micelle, the caseins are in direct contact via hydrophobic Van der Waals and hydrogen bonds but can also form bridges via calcium, calcium phosphate, or micellar calcium phosphate by means of their phosphoseryl residues [12]. Calcium phosphate contacts within the micelle are weakened by decreasing calcium content and pH [13]. A κ-casein surface layer stabilizes the casein micelle sterically and prevents further accumulation of caseins [14]. Acidification or hydrolysis after enzymatic treatment with the enzyme chymosin causes the κ-casein surface layer to lose its extended structure and the surface-modified micelles to lose their colloidal stability. The resulting coagulation leads to colloidal gel networks, which are used for the production of yogurt or cheese [3]. However, if the enzymatic treatment is carried out in the cold, the hydrolysis of κ-casein occurs more slowly but without loss of the colloidal stability of the casein micelles. By increasing the temperature again, the coagulation can be decoupled from the preceding hydrolysis, which opens a range of new process engineering possibilities to form targeted functionalized gelled casein structures [15,16]. Extrusion of cold-gelled casein micelles into a warm, calcium-rich coagulation bath resulted in micellar casein fibers that expanded to a constant swelling value in 1 N HCl and disintegrated under strongly basic conditions [7]. Confocal fluorescence microscopy images showed an internal network structure of the fiber, which was preserved even after acidification in 1 N HCl, but exhibited more compact strand structures in contrast to the fiber structure under neutral pH conditions [17]. The strands of the gel were a few µm thick and thus in the range of a few interconnected casein micelles. Fibers prepared with different concentrations of CaCl_2_ in the coagulation bath showed that calcium bridges within the fiber structure are important for stability [17,18]. Previous studies on milk gels have shown that with decreasing pH, calcium phosphate bonds are destabilized and the concentration of free calcium and phosphate increases [19].

In this study, we investigate the influence of acidic milieu conditions on the stability of the rennet gel network of micellar casein fibers. For this purpose, we use aqueous swelling media with pH values < 2, which we adjust with citric acid of different molarities. To understand the typical two-phase swelling process in acidic media, we precisely measure the increases in the fiber width and analyze the obtained swelling curves using simulations of a rate equation. 

## 2. Results and Discussion

**Figure 1:**
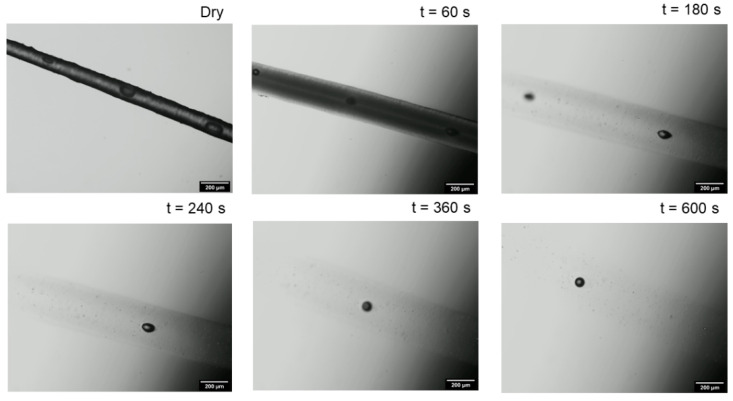
Sequence of microscopic images demonstrating the swelling of a regenerated casein fiber in acidic media, shown for 1 M citric acid as an example.

Micellar casein fibers are spun by extruding cold-renneted casein micelles through a nozzle into a warm coagulation bath. For the formation, high calcium concentrations > 50 mM must be used in the coagulation bath. Inside the casein micelles used as raw material for fiber production, calcium neutralizes the negative charge of caseins, bridges colloidal calcium phosphate with the phosphoserines of caseins, and forms inter-protein or intra-protein linkages between two phosphoserines [12]. Drastic acidification leads to the complete dissociation of the calcium phosphate contacts, but the integrity of the casein micelle remains intact [20]. To investigate this effect on fiber stability, we studied the influence of acidic media on the swelling of the casein matrix. For this purpose, dried casein fibers were placed in swelling media of different citric acid concentrations (0.125 M, 0.5 M, and 1 M), and the thickness increase was recorded as a function of time. Figure 1 shows an example of a sequence of microscopic images of a swelling fiber in 1 M citric acid. The fiber thickness increased more and more over time. Due to the solvent inflow, the contrast between fiber and medium decreased more and more until the contour of the fiber was no longer detectable after approx. 600 s of swelling. This is in contrast to the swelling in 1 N HCl, where, after initial swelling, a deswelling to an equilibrium swelling value took place [17].

**Figure 2:**
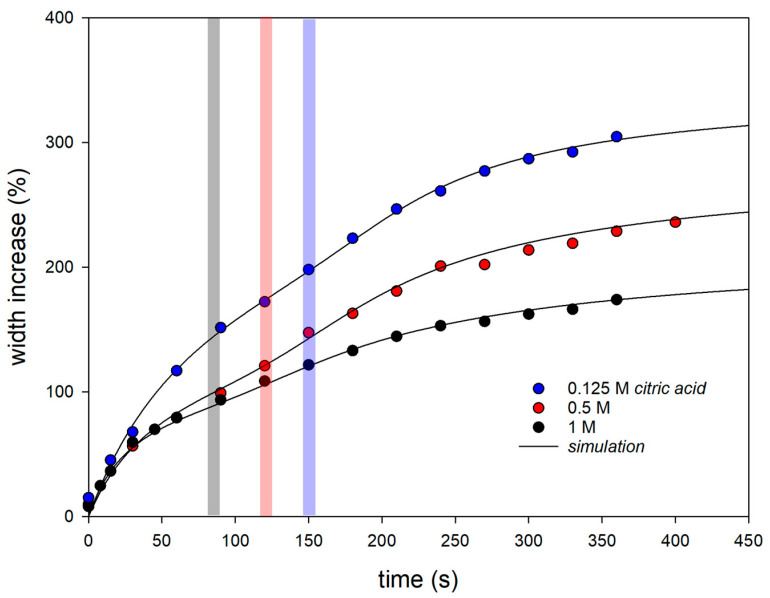
Swelling curves of casein fibers in citric acid with different molarities, obtained by averaging over all experimental data. Solid lines are simulations with Equation (8). The colored bars roughly indicate the beginning of the second swelling process.

Figure 2 shows the thickness increase in dried fibers in aqueous swelling media of citric acid with three different molarities. The values shown are mean values over all experimental data (three observations each of three different fibers). The solid lines correspond to simulations with a swelling model, which is discussed below. It can be clearly seen that the swelling process is biphasic and that the lower the molarity of the citric acid in the swelling medium, the stronger the swelling. The pH values of the swelling medium calculated from the molarities were pH 2.03 for 0.125 M citric acid, pH 1.72 for 0.5 M, and pH 1.57 for 1 M. Furthermore, it can be seen that the later that swelling process 2 starts, the weaker the acidity or higher the pH of the swelling medium (see the position of the bars in Figure 2). Previous fiber swelling studies in 1 N HCl have already indicated a pH transition of two different swelling processes that occurred only after a low pH was reached [17]. This observation is confirmed by the experiments in citric acid. The higher the molarity of citric acid in the swelling medium, the faster the critical pH in the fiber is reached, which causes the transition to swelling process 2. However, the swelling curves in HCl showed overshooting after the initial swelling followed by deswelling to an equilibrium value. For the analysis of the citric acid experiments, the swelling model must therefore be modified by a sign change (indicated by a curly bracket in Equation (8)) that turns a solvent outflow into an inflow for swelling process 2.

**Figure 3:**
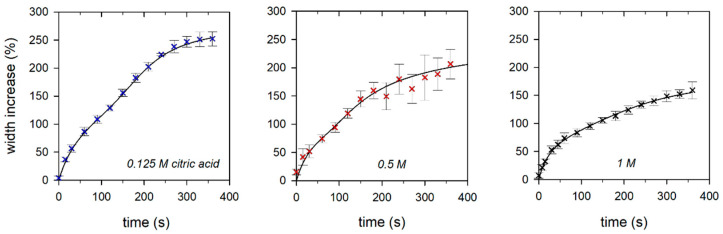
Swelling curves of individual micellar casein fibers in swelling media with different citric acid content. The error bars correspond to the standard deviation calculated from measured values at three different fiber positions.

For a more detailed analysis, the swelling values measured at three different positions of a single fiber were averaged for different times, plotted as the width increased, and analyzed with Equation (8). As a selected example, Figure 3 shows the increase in fiber width of one of the three fibers for each citric acid medium. It can also be clearly seen from the swelling data of individual fibers that the increase in fiber diameter basically follows a two-phase process.

**Figure 4:**
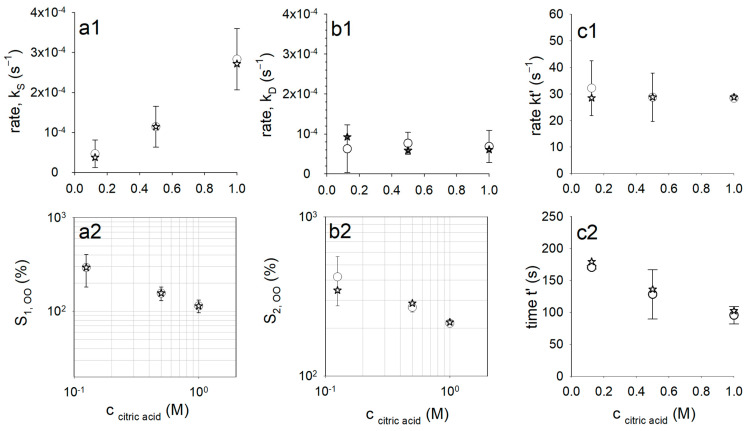
Parameter values of the kinetic swelling model (Equation (8)) as a function of the citric acid concentration for the simulations of the swelling curves in Figure 2 from all averaged swelling data (asterisks), as well as of single fibers (open circles) with standard deviations showing the variability of the swelling data from 3 different single fiber experiments. Values for the rate coefficient, k_S_ (**a1**), and maximum swelling value, S_1,∞_ (**a2**), of the first swelling process and for rate coefficient, k_D_ (**b1**), and maximum swelling value, S_2,∞_ (**b2**), of the second swelling process, as well as for rate, k_t’_ (**c1**), and characteristic time, t’ (**c2**) of the mono-exponential kinetics (Equation (6)).

The fiber diameter in 0.125 M citric acid initially increases strongly within the first 100 s and later more weakly, before increased swelling occurs again from approx. 150 s onwards. The transition from swelling process 1 to swelling process 2 shifts to shorter times with increasing acidity of the swelling medium, as was already the case with the swelling data averaged over all fiber experiments (compare with Figure 2). Furthermore, it can be clearly seen that the maximum swelling value achieved during the observation period decreases accordingly. The parameter values for the model simulations shown in Figure 3 (solid lines in Figure 3) are plotted as open circles in Figure 4.

**Figure 5:**
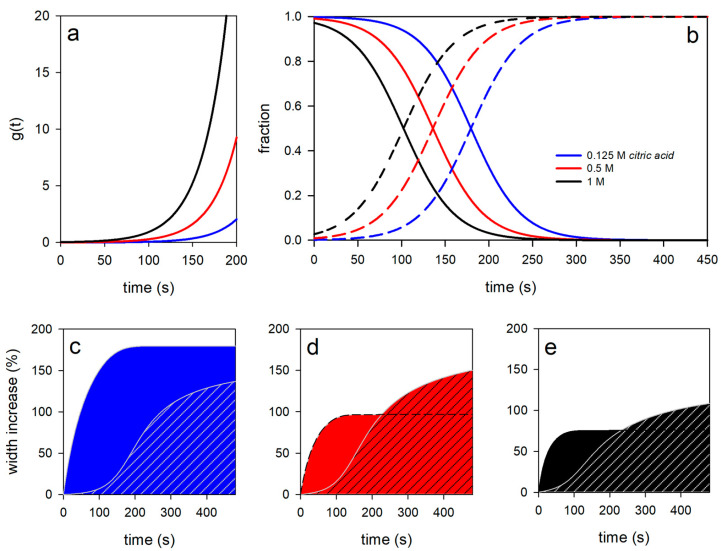
Results of the fiber swelling simulation with the swelling model Equation (8) as a function of the citric acid concentration. (**a**) Functions g(t), which describe the speed of acidification in the fiber; (**b**) shares of both gel types in the total swelling rate as a function of the swelling time; (**c**–**e**) calculated fractions of the swollen fiber structures for the renneted gel (filled area) and the acidified renneted gel (hatched) in the total swelling process.

The rate $k_{S}$ of initial solvent uptake of the dried fiber increases superlinearly with increasing molarity of the citric acid in the swelling medium (see Figure 4(a1)). However, as Figure 4(a2) shows, the maximum degree of swelling, $S_{1,\infty }$, associated with swelling process 1 decreases with increasing citric acid concentration. We observe a similar effect in the second swelling process for $S_{2,\infty }$ (Figure 4(b2)), while, in contrast, the corresponding swelling rate remains almost unchanged, with $k_{D}\approx 7\cdot 10^{-5}\;s^{-1}$ (Figure 4(b1)). The different swelling rates in processes 1 and 2 are the result of a structural change within the fiber, which takes place in the course of the pH reduction from a rennet gel to an acidified rennet gel. The higher the acidity of the swelling medium, the faster this process runs. We model this acid-induced gel transition using the ratios described by Equation (7) for the swelling process 1 or 2, which are calculated via an exponential function. The rate of the exponential function remains with $k_{t^{\prime }}\approx 30\;s^{-1}$ almost constant, while the characteristic time, *t*′, shifts to smaller and smaller values with increasing acid strength, as the simulation results in Figure 4(c1,c2) indicate. The calculated curves of g(t) are shown in Figure 5a, on the basis of which the time-varying proportions for processes 1 and 2 (Equation (7)) are determined. The calculated proportions of the rennet gel network decrease from 1 to 0 as the fiber acidifies, while the proportions of the acidified rennet gel network increase from 0 to 1. This took longer when the acid strength of the citric acid was lower, as can be seen from the number of times taken to reach the half values of the sigmoidal transition curves. The reason for the structural change is pH-dependent changes in the strength of the interactions between the caseins in the gel network of the fiber. For casein micelles, it has been shown that after the pH falls below 5.3, all micellar calcium phosphate is dissolved [21], or that all inorganic phosphate or calcium is in a solution below pH 5.3 or pH 3.5, respectively [22]. The loss of calcium-mediated stabilizing contacts leads to the increasing dissociation of the casein network. We have recently shown that the dissolution of calcium contacts in casein gel networks, more precisely in casein micro-particles, does not occur immediately but requires at least three medium exchange cycles of about 10 min from pH 11 to pH 1 and back [23]. However, chelating agents such as the citric acid or EDTA used here significantly increase the proportion of calcium and phosphate in the diffusible phase [24] without forming cross-links or interfering with gelation [25]. An increased swelling rate of casein micro-particles was indeed reported after the addition of citrate in the mM range at neutral pH [26].

As a result of changed interactions between the caseins, the casein solvent properties also change. In the course of acidification up to approx. pH 5.5, the solubility properties of the caseins increasingly worsen, which is reflected in an increase in the fluorine intensity of the aromatic amino acids and in the shift of the fluorescence maximum toward shorter wavelengths [27]. When the isoelectric point of the caseins is crossed at pH = 4.8, the caseins within the network adopt their most compact form due to the lowest electrostatic repulsion. In this pH range, the system becomes unstable due to a restructuring of the caseins, which makes, e.g., the interpretation of fluorescence data impossible, and dynamic moduli show a significant maximum [28]. Casein molecules aggregate under these conditions to form casein particles, which are very different from casein micelles in milk at neutral pH [29]. Stabilizing calcium phosphate contacts no longer exist because they are completely dissolved after acidification. Mainly Van der Waals and hydrophobic interactions, as well as hydrogen bonds, at this point hold the gel network together in this strongly acidic pH range [4]. If the pH in the fiber falls further to the pH values of the swelling media used, the solubility conditions of the caseins improve again due to the protonation of amino acids. In order to discuss the occurrence of the two different gel types and their swelling capacity in the course of the swelling experiment, their proportion is calculated for all three citric acid media as a function of time. For this purpose, Equation (8) was integrated over the product of the respective swelling rate and the corresponding ratio, $w_{i}$, according to

(1) $$S_{i=1,2}=\int_{t=0}^{t_{max}}w_{i}\cdot \frac{dS_{i}}{dt}dt\;$$

The swollen structure that can be attributed to the rennet gel network is indicated in Figure 5c by a curve with a filled area, while that for the acidified rennet gel is hatched. 

The fiber, which is dry at the beginning, swells hyperbolically during process 1, i.e., initially at a high rate and then at a decreasing rate in all three citric acid concentrations. Despite the higher rate coefficient $k_{S}$, the kinetics in 1 M citric acid are determined by a significantly smaller final swelling value of $S_{1,\infty }$ than in the swelling media with lower molarity. Due to the higher acid strength, the proportion of dissociated salt ions in the solvent increases. This decreases the solvent quality and probably causes the rennet gel network to shrink [30]. On the other hand, at the beginning of acidification at pH values above the isoelectric point, negative residual charges remain in the rennet gel when protein-bound calcium dissociates. Their greater proportion at higher acidity increases repulsion and probably widens the mesh size of the gel network, which could explain the increase in rate $k_{S}$ with increasing molarity of citric acid.

Swelling process 2 is also modeled via second-order swelling kinetics, with a hyperbolic swelling curve as a solution, according to Equation (4). In contrast to swelling process 1, however, the gel structure for swelling process 2 must first be formed during acidification. In rheological studies, a sigmoidal increase in the storage modulus was observed in the course of rennet-induced skim milk gel formation [31] as well as after acidification with glucono-δ-lactone for acidified milk gels [4]. Our simulations also provide sigmoidal functions for the evolution of the swollen structure of the acidified rennet gel (curves with shaded areas in Figure 5c–e). The swelling model (Equation (8)) is thus able to describe the formation of a new gel type and, in parallel, its swelling. As Figure 5c–e show, the inflection point, and thus the time of maximum swelling rate for process 2, shifts with increasing proton concentration from approx. 230 s at 0.125 M citric acid over 200 s (0.5 M) to 180 s (1 M). Since the rate coefficient, $k_{D}$ is independent of the citric acid concentration used (Figure 4(b1)), the effect is mainly based on the dotted ratio curves in Figure 5b, i.e., how fast the isoelectric point is passed and the transition to the second gel type is reached. At pH values below the isoelectric point in the acidic rennet gel network, both calcium and phosphate are completely dissociated, and an effect of the citric acid concentration on the swelling rate is no longer detectable.

**Figure 6:**
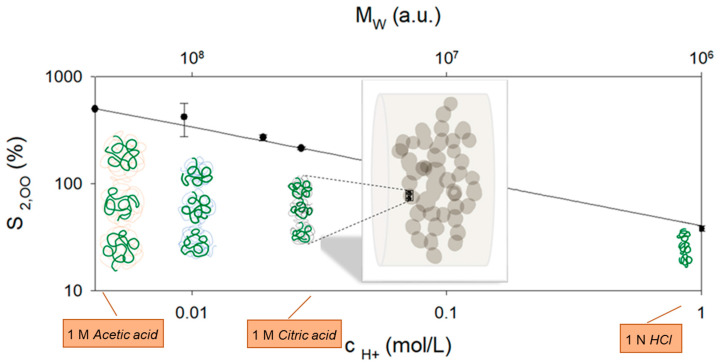
Scaling between the maximum achieved degree of swelling of the fiber and the proton concentration adjusted via various organic and mineral acids. Proposed acid-induced reduction in the molecular weight of the casein aggregates leading to shortening of the strands within the particle network (schematic representation of the particle network is reproduced from Thill et al., 2021 [7]).

However, the maximum degree of swelling $S_{2,\infty }$, and thus also the total degree of swelling of the fiber at the end of the kinetics, are dependent on the citric acid concentration. There is a reduced expansion with increasing proton concentration in the medium, similar to the swelling of the rennet gel network $S_{1,\infty }$ (see Figure 4(a2,b2)). While for the rennet gel at pH values above the isoelectric point, shrinkage can be explained with the decrease in the solvent quality due to dissociated calcium phosphate [30], this is no longer true for the acidic rennet gel at lower pH values. For the citric acid concentrations used, pH values far below the isoelectric point are reached at the end of the kinetics, so that the calcium phosphate of the gel network is completely dissociated in all three swelling media. Interestingly, there is a scaling behavior of the maximum degree of swelling with the proton concentration (see Figure 6), which is confirmed by measurements in HCl [17] and in 1 M acetic acid (swelling data not shown).

In detail, the fit gives scaling as $S_{2,\infty }\sim c_{H^{+}}^{-0.46}$. After acidification with different concentrations of acetic acid (1 M and above), a percentage of casein was observed to dissolve out of the acid gel after times > 30 min and increased with acetic acid concentration [32]. This is an indication that the sum of the remaining stabilizing interactions (Van der Waals, hydrophobic, and H-bridges) decrease with decreasing pH, thus reducing the size of interconnected casein aggregates in the network.

A plausible explanation for the decreasing maximum degree of swelling with increasing proton concentration could therefore be the volume requirement of the remaining aggregated casein molecules within the gel network at the moment of dissolution. This can be described by a critical concentration of the polymer chains, which depends on the molecular weight and the conformation of the casein aggregates. In their native state, caseins exist in an open and flexible (native, unfolded) conformation [8]. In contrast, at the isoelectric point, caseins collapse into dense coils. Since the solubility conditions improve again below the isoelectric point and the charges are screened by dissociated calcium phosphate due to the high ionic strengths, a random coil conformation of the interconnected caseins in the gel network can be assumed at the end of the swelling kinetics. During swelling to dissolution, the distance between neighboring casein aggregates within the fiber network continues to increase.

At the critical concentration, c*, where polymer chains lose contact with each other, the concentration of monomer, *N*, within a dilute conformation of size, *R*, is equal to the concentration in the medium (here in the strands of the fiber), and it holds $c^{*}\approx N/R_{dilute}^{3}$ [33]. When casein fibers swell to a stable final value of around 50% of the initial value under neutral pH conditions in a simple swelling process, the fiber network is slightly stretched in the fiber direction [7]. If the increase is 100–300%, as in the experiments shown here, after acidification to pH < 2.0, it can be assumed that the strands stretch perpendicular to the fiber direction until the casein aggregates lose contact. A corresponding schematic is shown in Figure 6, which is based on a fluorescence microscope image from [7]. For this situation, we define analog to $c^{*}$ a critical length, $L^{*}$, within the strands:

(2) $$L^{*}\sim \sqrt{\frac{1}{c^{*}}}\;$$

In a θ solvent, the end-to-end distance of an ideal coil is $R_{0}=b\cdot N^{\frac{1}{2}}$ (*b* corresponds to the Kuhn monomer size), and for a good solvent, the Flory end-to-end distance $R_{F}=b\cdot N^{0.588}$ can be used to derive the corresponding scale laws between c* and the molecular weight of the chain conformations [34]. If one assumes that the rate of acid-induced dissociation of casein aggregates is proportional to the proton concentration, similar to the acid hydrolysis of caseins (also a second-order reaction) [35], there is also a corresponding scale correlation between molecular weight and maximum swelling degree or critical length ($L^{*}\equiv S_{2,\infty }\sim M_{W}^{0.46}$, upper horizontal scale, Figure 6). Such proportionality would result in casein aggregates, which have a similar conformation to a neutral polymer in a good solvent. The volume expansion of different molecular weights with coil conformation and a constant molecular weight chain (green, e.g., a casein monomer) contained therein are shown in Figure 6 to explain the maximum swelling shortly before dissolution.

## 3. Conclusions

Gel networks produced from casein micelles form the basis of many milk products, such as yogurt or cheese. While the formation processes for acidified and rennet milk gels are well studied at the molecular level, there are far fewer mechanistic studies dealing with the processing of existing milk gels. Here, we demonstrate with swelling experiments and simulations that one can very accurately and reproducibly study the transition from a rennet casein gel to an acidified rennet casein gel. In contrast to one-step swelling in water under neutral pH conditions, we observe a two-phase swelling process in both organic and mineral acids, which we explain with the transition from a rennet casein gel type to an acidified rennet gel type via an instability/restructuring at the isoelectric point. We describe the swelling kinetics by second-order rate equations and time-dependent ratios of the swelling rates of both gel types. The rate coefficient of the first swelling process increases with the increasing acidity of the swelling medium due to the stronger dissociation of stabilizing calcium contacts and the solvation of residual charges on casein. However, the accompanying increase in ionic strength results in a decrease in solvent quality, which leads to a reduced expansion of the caseins and a decrease in the equilibrium swelling value for the first swelling process. Below the isoelectric point of the caseins, non-ionic casein–casein contacts stabilize the acidified network, and the swelling rate of the network is then independent of the molarity of the citric acid in the swelling medium. In the strongly acidic pH range at the end of the kinetics, the maximum expansion of the fiber is a result of the swelling of this gel type without stabilizing calcium phosphate links. The final fiber thickness decreases with increasing acidity and is proportional to $S_{2,\;max}\sim c_{H^{+}}^{-0.46}.$ We assume that this is due to a reduction in the molecular weight of the casein aggregates, which are present as coils in good solvents at pH < 2, and a corresponding shortening of their length where overlaps still occur in the strands perpendicular to the fiber axis. Future studies should aim to directly measure the pH in the swelling fiber. Furthermore, we intend to produce more fibrous material in order to investigate the size of the aggregates of a dissolving fiber, e.g., by gel electrophoresis or light scattering.

## 4. Materials and Methods

### 4.1. Preparation of the Raw Material

Casein micelles were isolated from fresh whole milk from a local dairy farm (Soerser Milchkännchen, Aachen, Germany). After fat separation at 3000× RCF for 20 min, the milk was then centrifuged at 70,000× RCF for 60 min in an OptimaXP-80 ultracentrifuge (Beckman Coulter GmbH, Krefeld, Germany) to separate the micellar casein protein form the rest of whey proteins. The casein pellet was resuspended in simulated milk ultrafiltrate (SMUF) by stirring for 2 h at 37 °C after the supernatant was removed. The SMUF buffer was prepared according to Dumpler, 2017 [36]. For the preparation of the SMUF buffer, the corresponding salts were added successively to 1 L of deionized water under constant stirring at room temperature in the concentration and order shown in Table S1. To prevent microbial growth, 0.05% (*w*/*w*) sodium azide (99%, Carl Roth, Karlsruhe, Germany) was added to the sample. The casein micelle concentration was determined gravimetrically and adjusted to 5% (*w*/*w*) by dilution with SMUF. To achieve a stable solution by cold renneting, the casein micelles solution was incubated with 100 IMCU L-1 fermentation-produced chymosin (CHY-MAX M, Chr. Hansen A/S, Hoersholm, Denmark) at 5 °C for 24 h. 

### 4.2. Fiber Production

The enzyme-treated casein micelle solution stored at a low temperature was extruded into a warm, calcium-rich coagulation bath (T = 60 °C, $c_{CaCl_{2}}$ = 100 mM) using a syringe pump (dV/dt = 0.4 mL/min) and a dosing needle with an inner diameter of 0.8 mm. The fibers obtained were picked up with tweezers and, after rinsing in deionized water, placed on a rack in such a way that fiber pieces with a length of 3 cm could be air-dried without contact with surfaces. 

### 4.3. Swelling Experiments 

The fibers were fixed on a Petri dish and the swelling process in different acids was investigated with an inverted microscope (Nikon Eclipse TE300, Tokyo, Japan). In detail, swelling experiments were carried out in 1 M, 0.5 M, and 0.125 M citric acid, and, for comparison, also in 1 M acetic acid, in 1 N HCl, and in water. For this purpose, a video of the swelling process was recorded with a camera installed on the microscope (AMscope Camera, Irvine, CA, USA). For each swelling medium, fiber thicknesses were determined as a function of time ($d_{t}$) on three different fiber preparations and for each fiber at three points, using ImageJ software. After averaging, the increase in fiber thickness was calculated according to

(3) $$width\;increase\;\left(\%\right)=\left(\frac{\langle d_{t}\rangle }{\langle d_{0}\rangle }-1\right)\cdot 100$$

using the fiber diameter of the dry fiber $d_{0}$.

### 4.4. Swelling Model

The swelling of dry micellar casein fibers in aqueous media at neutral pH can be modeled by second-order swelling kinetics [17], which method was used by Schott, 2006, to describe the swelling of gelatin and cellulose [37].

(4) $$S=\frac{k_{S}\cdot S_{\infty }^{2}\cdot t}{1+k_{S}\cdot S_{\infty }^{2}\cdot t}$$

Here, *S* is the solvent uptake at time, *t*, and $S_{\infty }$ is the swelling value reached at time t→∞. To model the two-phase swelling/deswelling in HCl, the rate equation belonging to Equation (4) can be used for both swelling processes:

(5) $$\frac{dS}{dt}=k_{S}\cdot {\left(S_{\infty }-S\right)}^{2}$$

Here, the swelling rate $\frac{dS}{dt}$ is proportional to the specific swelling rate$\;k_{S}$, and the square of the remaining swelling capacity$\;{\left(S_{\infty }-S\right)}^{2}$. The acidification of the fiber caused by solvent inflow was modeled by an exponential function with rate $k_{t}$ and a delay time *t*′ according to:

(6) $$g\left(t\right)=e^{\left(t-t^{\prime }\right)\cdot k_{t}}$$

From this, the dynamic ratios for swelling (index 1) and deswelling (index 2) in the total swelling are calculated via

(7) $$w_{1}=\left(\frac{1}{1+g\left(t\right)}\right);w_{2}=\left(\frac{g\left(t\right)}{1+g\left(t\right)}\right)$$

According to Thill et al., 2022 [17], the swelling rate for the overall process is then defined via

(8) $$\frac{dS}{dt}=w_{1}\cdot k_{S}\cdot {\left(S_{1,\infty }-S\right)}^{2}\overset{+}{\overbrace{-}}w_{2}\cdot k_{D}\cdot {\left(S_{2,\infty }-S\right)}^{2}$$

### 4.5. Statistical Analysis

Each swelling experiment was performed on 3 different fiber preparations. The swelling value of a fiber was obtained from 3 measurements at different locations on the fiber. A sensitivity analysis was performed using the Stella 1.6 program (iseesystems.com, accessed on 21 February 2019, Lebanon, NH, USA) to optimize the swelling kinetic simulations. Error bars of the simulated parameter values are based on the variability due to the triple determination on three different samples.

## Data Availability

Data presented within the article.

## Supplementary Materials

The following supporting information can be downloaded at: https://www.mdpi.com/article/10.3390/gels9070538/s1, Table S1: Preparation of SMUF solution.
