# Supplementary material for: Regenerated Fibers from Rennet-Treated Casein Micelles during Acidification
Source: Gels. 2023 Jul 3;9(7):538. doi: 10.3390/gels9070538 (PMC10378896; doi:10.3390/gels9070538)
Supplement: Supplementary file 1 [file gels-09-00538-s001.zip › gels-2452521-supplementary.pdf]

Supplementary Material: Regenerated Fibers from Rennet-Treated Casein Micelles during Acidification

Ronald Gebhardt [1,*] and Novin Darvishsefat [1]

Table S1. Preparation of SMUF solution according to Dumpler et al. 2017 [1].

Number	Concentration (g/L)	Component	Supplier
1	0.933	KH_2PO_4	Acros organics
2	0.867	K_2HPO_4	Acros organics
3	0.7	$K_3C_6H_5O_7.H_2O$	Alfa aesar
4	1.2	$Na_3C_6H_5O_7.2H_2O$	Acros organics
5	0.6	$Mg_3(C_6H_5O_7)_2.9H_2O$	Sigma Aldrich
6	0.057	$C_6H_8O_7.H_2O$	VWR chemicals
7	0.633	KCL	Merck
8	0.167	NaCl	VWR chemicals
9	0.2	K_2SO_4	Acros organics
10	1.233	$CaCl_2.2H_2O$	VWR chemicals

[1] Dumpler, J., Kieferle, I., Wohlschläger, H., & Kulozik, U. Milk ultrafiltrate analysis by ion chromatography and calcium activity for SMUF preparation for different scientific purposes and prediction of its supersaturation. *International Dairy J.* **2017**, *68*, 60–69.
